# Dual-Task Walking in Challenging Environments in People with Stroke: Cognitive-Motor Interference and Task Prioritization

**DOI:** 10.1155/2018/7928597

**Published:** 2018-05-02

**Authors:** Celine Timmermans, Melvyn Roerdink, Thomas W. J. Janssen, Carel G. M. Meskers, Peter J. Beek

**Affiliations:** ^1^Department of Human Movement Sciences, Faculty of Behavioural and Movement Sciences, Vrije Universiteit Amsterdam, Amsterdam Movement Sciences, Van der Boechorststraat 9, 1081 BT Amsterdam, Netherlands; ^2^Amsterdam Rehabilitation Research Center, Reade, Overtoom 283, 1054 HW Amsterdam, Netherlands; ^3^Department of Rehabilitation Medicine, VU University Medical Centre, Amsterdam Movement Sciences, De Boelelaan 1118, 1081 HZ Amsterdam, Netherlands

## Abstract

Cognitive-motor interference may contribute to the risk of falling in people with stroke, as may be the associated phenomenon of inappropriate task prioritization. Examining dual-task walking could provide valuable insights as to how to best evaluate and treat walking in people with stroke. This study aimed to examine the effect of different walking environments on cognitive-motor interference and task prioritization in dual-task walking in people with stroke. Using a repeated-measures design, cognitive-motor interference and task prioritization were assessed in 30 stroke survivors, while walking in a plain environment and in two challenging environments that were enriched with either stationary physical context or suddenly appearing projector-augmented context. All three walking environment conditions were performed with and without a concurrent serial-3 subtraction task. We found stronger cognitive-motor interference for the two challenging environments than for the plain walking environment. Cognitive-motor interference did not differ between challenging walking environments, but task prioritization did: motor performance was prioritized more in the environment with physical context than in the environment with projector-augmented context and vice versa for cognitive-task performance. In conclusion, walking environment strongly influenced cognitive-motor interference and task prioritization during dual-task walking in people with stroke.

## 1. Introduction

Walking is a semiautomatic activity that is often combined with talking, calling, texting, and other attention-demanding activities. When exposed to challenging walking environments, such as a busy crossroad or cluttered terrain, walking itself becomes more attention demanding. Combining walking with concurrent cognitive tasks will typically degrade overall task performance (i.e., cognitive and motor performances combined), as evidenced by a lower walking speed, poorer walking-adaptability performance (e.g., obstacle avoidance), and/or poorer cognitive-task performance. This reduction in overall task performance during dual-task walking is called cognitive-motor interference [1–3]. Patients with walking limitations, including patients with stroke, suffer from greater cognitive-motor interference than healthy adults [4], which increases their risk of falling [5].

Several studies reported improvements in dual-task walking speed after cognitive dual-task gait training in people after stroke [6, 7]. However, still little is known about dual-task walking in challenging walking environments. A relevant aspect of dual-task walking is task prioritization, which may alter as walking environments become more challenging. For people with stroke this has been examined in laboratory treadmill studies, where an obstacle-avoidance task was performed with and without an attention-demanding cognitive task [8, 9]. In these studies, wooden obstacles were suddenly presented under high time-pressure demands (i.e., within a single stride) with considerable physical repercussions of failure (e.g., stumbling, tripping). The results showed that obstacle-avoidance performance was prioritized over cognitive-task performance, the so-called “posture-first principle.” That is, obstacle-avoidance success rates were similar with and without the cognitive dual task while cognitive-task performance decreased significantly with dual-tasking, particularly so for the obstacle-negotiation stride [8, 9].

A very common adaptation when encountering a challenging walking environment, or when performing a dual task while walking, is to walk slower [10, 11]. However, this walking speed adaptation was precluded in those laboratory studies because walking speed was kept constant experimentally. Consequently, our understanding of dual-task walking of people with stroke in challenging environments is still incomplete, which is unfortunate given its association with common causes and circumstances of walking-related falls [12]. For instance, inappropriate task prioritization may occur when people give priority to a telephone conversation while maintaining their walking speed and stumble over an obstacle while doing so. Examining dual-task walking, without constraining walking speed, which is more similar to walking in daily life situations, could provide a better understanding of dual-task interference and task prioritization, which may yield valuable suggestions as to how to best evaluate and treat (adaptive) walking in people with stroke.

The aim of the present study was to examine the effect of walking environment on cognitive-motor interference and task prioritization in dual-task walking in people with stroke without constraining walking speed. In particular, we contrasted a plain overground walking environment (10 Meter Walking Test [10MWT]) with two different challenging overground walking environments with context (e.g., stepping targets, obstacles), demanding adaptive walking. The two challenging walking environments differed with regard to the type of context: a stationary physical context versus suddenly appearing projector-augmented visual context. The latter context was included as projector-augmented walking-adaptability treatment is increasingly used as part of rehabilitation after stroke [9, 13–15]. We expected stronger cognitive-motor interference for the two challenging walking environments, as reflected in lower walking speeds, poorer walking-adaptability performance, and/or poorer cognitive-task performance. With regard to task prioritization, we additionally expected superior walking-adaptability performance combined with inferior cognitive-task performance (i.e., posture-first principle) for the walking environment with physical context and vice versa for the environment with projector-augmented context, given the less salient repercussions of walking-adaptability failures in the environment with projector-augmented context.

## 2. Methods

This study was part of an ongoing clinical trial that was designed to compare the effects of two interventions for improving walking speed and walking adaptability in people with stroke [16], which was approved by the Medical Ethical Reviewing Committee of VU University Medical Centre (Amsterdam, Netherlands, protocol number 2013/53, and Central Committee on Research Involving Human Subjects, CCMO, protocol number NL 42461.029.13).

### 2.1. Participants

Thirty-three stroke survivors were recruited from the inpatient and outpatient population of Rehabilitation Center Reade (Amsterdam, Netherlands). Inclusion and exclusion criteria are detailed in Timmermans et al. (2016) [16]. Three participants were excluded because of missing data due to technical errors. The remaining 30 participants (mean ± SD; age: 55 ± 12 years, height: 173 ± 9 cm, and body mass: 77 ± 13 kg, 17 males) had a first-ever stroke ≥ 3 months before study entrance (53 ± 73 months), all infarction, and a Functional Ambulation Categories (FAC) score ≥ 4 (FAC 4/5, *n* = 2/28), were clinically diagnosed with hemiparesis (15 left hemiparesis), suffered from walking and/or balance deficits (13 used an assistive device) as confirmed by a physician, had a low executive functioning score assessed with the Trail Making Test (TMT) (B/A ratio: 2.39 ± 0.83) [17], and had no moderate or severe cognitive impairments as indicated by a Mini-Mental State Examination (MMSE) score < 21 (MMSE: 28 ± 2 points). All participants provided written informed consent before the start of the clinical trial.

### 2.2. Procedure and Set-Ups

**Figure 1:**
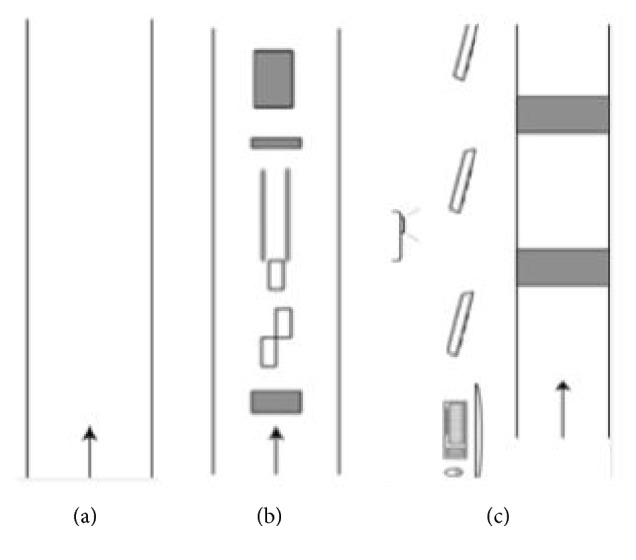
Schematic representations of the three walking environments. (a) Plain walking environment. (b) Challenging walking environment with stationary physical context (challenging-physical; comprising three stepping targets, a 2-m tandem-walking path, and three obstacles). (c) Challenging walking environment with suddenly appearing projector-augmented context (challenging-projected; comprising position-dependent and gait-dependent obstacles).

As part of the baseline measurements of the clinical trial, participants walked in three different environments (i.e., a plain walking environment and two challenging walking environments enriched with either stationary physical context (challenging-physical) or suddenly appearing projector-augmented context (challenging-projected)), all with and without a concurrent cognitive task. This resulted in six walking conditions, which were performed in randomized order. For the plain condition, the standard 10 Meter Walking Test (10MWT) [18] was performed three times at a self-selected comfortable walking speed (Figure 1(a)). The challenging-physical condition comprised a 10MWT with physical objects on the walking path, including three obstacles (at 2.0 m, 7.5 m, and 9.0 m and of length × width × height 9.0 × 20.0 × 4.5 cm, 4.5 × 20.0 × 9.0 cm, and 33.0 × 21.0 × 11.5 cm, respectively), a tandem-walking path (from 4.5 m to 6.5 m with a width of 20 cm), and three stepping targets (participants' shoe length + 4 cm by shoe width + 4 cm) (Figure 1(b)). This challenging-physical condition was also performed three times at a self-selected comfortable walking speed; in this condition, participants were instructed to step over the obstacles and step onto the targets and in-between the tandem-path lines. The challenging-projected condition was conducted with the Interactive Walkway (IWW, Technology4Science, Vrije Universiteit Amsterdam, Netherlands) [19, 20], a 6.6 × 0.9 m walkway instrumented with multiple Microsoft Kinect for Windows sensors, and a projector to present obstacles (projected red rectangles of 0.4 × 0.9 m) in both a gait-dependent (i.e., one obstacle at a predicted foot-placement position appearing two steps ahead) and a position-dependent (i.e., one obstacle at an unpredictable but predefined position appearing when a participant's ankle was within 2 m from that obstacle) manner (Figure 1(c)). This challenging-projected condition was performed 10 times (including three dummy trials without obstacles, to retain unpredictability), again at a self-selected comfortable walking speed. Participants were instructed to step over the obstacles.

The concurrent cognitive task was a serial-3 subtraction task, counting backwards out loud. The number to start with was varied to avoid task familiarization. Participants practiced this subtraction task for 30 s while sitting. During all dual-task conditions participants were instructed to perform the tasks simultaneously and as effectively as possible at a self-selected walking speed. In addition, a 60 s subtraction task was performed while sitting in order to be able to determine the effects of walking environment on cognitive-motor interference (i.e., using sitting as the single-task reference for cognitive-task performance, see Outcome Measures). This 60 s seated subtraction task was randomized with the six walking conditions.

### 2.3. Outcome Measures

The effect of walking environment on cognitive-motor interference and task prioritization during dual-task walking was examined using the following dependent variables: (i) walking speed for all walking conditions (m/s, averaged over repetitions), (ii) a cognitive-task performance score for sitting and dual-task walking conditions (the number of correct subtractions per second; n/s, averaged over repetitions), and (iii) a walking-adaptability performance score for the challenging-physical and challenging-projected conditions (range 0–10). For the challenging-physical condition, this walking-adaptability performance score was the sum of subscores obtained for obstacle avoidance, tandem walking, and targeted stepping, averaged over the three repetitions. To be classified as a successfully avoided obstacle, both feet had to stay clear of the obstacle without stepping next to it, circumduction of the hip, or hitting the obstacle (one point per successfully avoided obstacle, with a maximum of three points). For successful targeted stepping, the whole foot had to be placed within the target without allowing intermediate steps (one point per successfully hit target, with a maximum of three points). Because the total number of steps for tandem walking was expected to vary among participants (5.41 ± 1.77 steps, according to the results), we categorized successful tandem walking based on the percentage of correct steps within the narrow-walking path: one point for 0–25% correct steps, two points for 26–50% correct steps, three points for 51–75% correct steps, and four points for 76–100% correct steps. For the challenging-projected condition, the walking-adaptability performance score was the sum of the points received for the first 10 obstacles in order to obtain the same scoring range as for the challenging-physical condition (range 0–10). To be classified as a successfully avoided obstacle, both feet had to be placed outside the area of the projected obstacle (i.e., no overlap of shoe and obstacle; one point per obstacle). For the challenging-projected condition, walking speeds and the number of correct subtractions were averaged over the trials involving these 10 obstacles (i.e., excluding dummy trials). Walking-adaptability performance was scored offline manually by two independent observers through visual inspection of sagittal video recordings and averaged in case of discrepancies (i.e., overall agreement in walking-adaptability score between the observers was 77% and 90% for challenging-physical and challenging-projected conditions, respectively).

The cognitive-motor interference during dual-task walking was quantified using the average of the respective dual-task effects of walking speed, the walking-adaptability performance score, and the cognitive-task performance score (with sitting as single-task reference), that is, motor (walking speed, walking adaptability) and cognitive scores combined to reflect overall task performance. Following Kelly et al. (2010), dual-task effects were defined as 100%  *∗* (dual-task performance minus single-task performance)/single-task performance [21] for walking speed, walking-adaptability performance scores, and cognitive-task performance scores. A negative cognitive-motor interference score indicates overall poorer dual-task than single-task performance, with more negative values indicating a stronger cognitive-motor interference.

### 2.4. Statistical Analysis

The effect of walking environment on cognitive-motor interference was examined using Friedman's ANOVA with within-subject factor environment (3 levels: plain, challenging-physical, and challenging-projected), using Wilcoxon signed-rank tests for post hoc analyses. Task prioritization across the walking environments was evaluated by comparing (1) the dual-task effect on both motor tasks (walking speed and walking-adaptability performance, using two-way repeated-measures ANOVAs with within-subject factors dual-tasking [2 levels: with and without serial-3 subtraction task] and environment [3 levels: plain, challenging-physical, and challenging-projected and 2 levels: challenging-physical and challenging-projected, respectively]) and (2) the dual-task effect on the cognitive task for each environment using a one-way repeated-measures ANOVA on cognitive-task performance score (4 levels: single-task reference condition, plain, challenging-physical, and challenging-projected). Task prioritization was determined by combining the findings of 1 and 2. Paired-samples *t*-tests were used for post hoc analyses of significant main effects or interactions involving the factor environment. Significant effects are reported (*p* < .05) as well as tendencies (.05 < *p* < .075). Effect sizes are represented by partial eta squared values (partial *η*^2^) and for post hoc tests with correlation coefficients (*r*).

## 3. Results

### 3.1. Cognitive-Motor Interference

**Figure 2:**
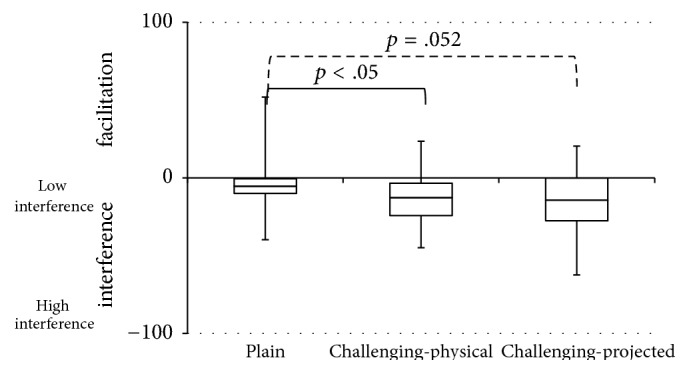
Boxplots of cognitive-motor interference scores for the plain walking environment and the two challenging walking environments with either stationary physical context (challenging-physical) or suddenly appearing projector-augmented context (challenging-projected). Negative values indicate poorer dual-task than single-task performance (i.e., cognitive-motor interference). One extreme outlier (223% in challenging-projected) was omitted from the figure. Significant effects (*p* < .05) are represented by solid lines and tendencies (.05 < *p* < .075) are represented by dashed lines.

Cognitive-motor interference scores are depicted in the boxplots of Figure 2. A significant effect of environment was observed (*χ*^2^(2) = 7.80, *p* < .05), with significantly stronger interference for the two challenging walking environments than for the plain environment (challenging-physical: *Z* = −2.09, *p* < .05, *r* = −0.27; challenging-projected: *Z* = −1.94, *p* = .052, *r* = −0.25), in the absence of a significant difference between the challenging-physical environment and the challenging-projected environment (*Z* = −0.01, *p* = .99, *r* = −0.001).

### 3.2. Task Prioritization

**Figure 3:**
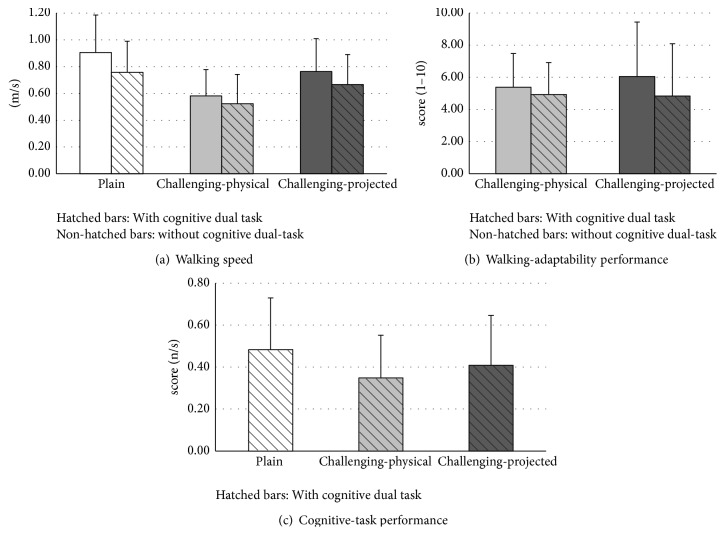
Representation of walking speed (a) for the six walking conditions (plain, challenging-physical, and challenging-projected, all with and without cognitive task), walking-adaptability performance score (b) for the two challenging walking environment conditions (challenging-physical and challenging-projected, with and without cognitive task), and cognitive-task performance score (c) for the three walking environments (plain, challenging-physical, and challenging-projected). Error bars represent the standard deviations.

In Figure 3 and Table 1, the effects of environment with and without dual-task walking are depicted for the dependent variables walking speed, walking-adaptability performance score, and cognitive-task performance score, as detailed below.

For walking speed, significant main effects of dual-tasking and environment were observed, as well as a significant interaction. Walking speed was lower with (0.65 ± 0.21 m/s) than without (0.75 ± 0.23 m/s) dual-tasking and differed significantly across all environments, decreasing from the plain (0.83 ± 0.24 m/s) via the challenging-projected (0.72 ± 0.24 m/s) to the challenging-physical (0.55 ± 0.20 m/s) walking environment (all *t*(29) > 5.20, *p* < .01, *r* > 0.69). Post hoc analysis for the interaction showed significantly lower walking speeds with than without the subtraction task for all walking environments (all *t*(29) > 2.28, *p* < .05, *r* > 0.39; for values see Table 1 and Figure 3), but the difference in walking speed was smaller for the challenging-physical (0.06 ± 0.14 m/s) than for the plain condition (0.15 ± 0.16 m/s; *t*(29) = 3.00, *p* < .01, *r* = 0.49), in the absence of significant differences between the plain and challenging-projected walking environments (0.11 ± 0.10 m/s; *t*(29) = 1.44, *p* = .16, *r* = 0.26) and between the challenging-physical and challenging-projected walking environments (*t*(29) = −1.76, *p* = .09, *r* = 0.31).

For walking-adaptability, a significant main effect of dual-tasking was observed, with lower scores with (4.88 ± 2.39 points) than without (5.72 ± 2.48 points) dual-tasking. Adaptability scores did not vary systematically with environment, but a tendency to an environment by dual-tasking interaction was observed, reflecting a smaller decline in walking-adaptability performance with dual-tasking in the challenging-physical environment (for values see Table 1 and Figure 3).

For cognitive-task performance, a significant main effect of environment was found. Post hoc analysis showed that the number of correct subtractions per second differed significantly across all environments, decreasing from the plain walking environment to the challenging-projected and challenging-physical walking environments (all *t*(29) > 2.71, *p* < .05, *r* > 0.45; for values see Table 1 and Figure 3). Note that for the two challenging environments the scores were significantly lower than in the 60 s single-task reference condition (all *t*(29) > 2.10, *p* < .05, *r* > 0.36).

## 4. Discussion

The aim of this study was to assess the effect of walking environment on cognitive-motor interference and task prioritization in dual-task walking in people with stroke. As expected, walking environment clearly affected cognitive-motor interference, with stronger interference for the two challenging walking environments than for the plain walking environment (Figure 2). Cognitive-motor interference did not differ between the two challenging walking environments, indicating a similar decline in overall task performance for dual-task walking. However, task prioritization clearly differed between the two challenging environments, as evidenced by reciprocal patterns in the magnitude of the differences in walking speed, walking-adaptability performance scores, and cognitive-task performance scores between single-task and dual-task conditions (Table 1 and Figure 3). That is, changes in walking speed and walking-adaptability performance scores were smaller with stationary physical context than with suddenly appearing projector-augmented context and vice versa for changes in cognitive-task performance scores. This indicates that motor-task performance was prioritized more with physical context than with projector-augmented context, at the expense of cognitive-task performance, which was prioritized less with physical context than with projector-augmented context. Thus, participants adhered more to the posture-first principle when walking in environments enriched with physical context than in environments enriched with projector-augmented context.

The main difference between the two challenging walking environments was the type of context, physical versus projector-augmented. As a result, the consequence of failure in walking-adaptability performance was more salient in the environment with physical context than in the projector-augmented environment. Participants clearly felt when physical obstacles were hit, and there was the actual probability of falling if the obstacle was contacted. In contrast, hitting a projector-augmented obstacle could only be perceived visually. The two environments thus strongly differed in penalty and feedback with regard to failures in walking-adaptability performance, thereby affording task-prioritization differences. Besides the difference with regard to type of context, the two environments also differed with regard to the time pressure for making walking adjustments (low for stationary physical context, high for suddenly appearing projector-augmented context). With projector-augmented context participants had to continuously monitor the environment for suddenly appearing obstacles and, when detected, a fast step adjustment was required. In this case, task prioritization likely varied throughout a trial [8, 9], placing strong demands on participants' task switching abilities. People with stroke generally have difficulty with task switching [22], which was also the case in the current sample as evidenced by the low TMT B/A ratios. Finally the difference in the amount of “clutter” (higher for physical context, lower for projector-augmented context) may have influenced task prioritization, because with physical context almost every step needed to be adjusted to obstacles, stepping targets, and the narrow-walking path, whereas with suddenly appearing projector-augmented context maximally two adaptations per trial were required. A greater amount of clutter in the walkway thereby required more attention-demanding step adaptations, which may thus have contributed to the poorer cognitive-task performance in the environment with physical context than in the projector-augmented environment.

Future studies are recommended to determine the relative contribution of the above-mentioned factors affecting task prioritization using systematic comparisons. For example, the consequence-of-failure factor could be studied by adding direct feedback on walking-adaptability performance to projector-augmented context (e.g., a beep when a projector-augmented obstacle is hit). To single out the effect of suddenly appearing context one could contrast the projector-augmented walking environments with walking environments containing the same, but continuously present, obstacles. To single out the effect of amount of clutter, one could systematically vary the amount of clutter in the walkway. The so-obtained insights could then be used to optimize assessments of cognitive-motor interference and task prioritization during walking. Interactive walkway assessments, as used for the projector-augmented conditions in the current study, seem well suited for such an endeavour because of its possibilities to standardize presentation of context in the walking environment in a movement-dependent manner and to objectify gait-environment interactions based on 3D kinematics [20]. Moreover, because of its 3D kinematics, this system could be useful in examining cognitive-motor interference during the different phases of obstacle crossing, as recently described in the study of Shafizadeh et al. (2017) [23], yet without constraining walking speed as in the studies of Smulders et al. (2012) and van Ooijen et al. (2015) [8, 9].

A limitation of this study was that the two observers that scored walking-adaptability performance were not blind to the condition (with or without cognitive task). Moreover, the single cognitive task while sitting was only performed once, in a 60 sec trial, rather than providing at least 3 trials as with the other tasks. These limitations notwithstanding, the current study is the first to address cognitive-motor interference and task prioritization in dual-task walking in plain and challenging environments in people with stroke. Two main results with practical significance were obtained. First, cognitive-motor interference becomes stronger in more challenging walking environments. And second, task prioritization during dual-task walking varies with the nature and type of context used to enrich walking environments. One should be aware of such effects when enriching treatment environments with context, as it could potentially result in inappropriate task prioritization. Interesting in that regard will be the results of the RCT of Timmermans et al. (2016) [16], in which two different treatment programs with challenging environmental context are compared: one with stationary physical context (FALLS program developed by Van Duijnhoven et al. (2012) [24]) and one with suddenly appearing projector-augmented context (C-Mill therapy). Based on the findings of the current study, the physical context used in the FALLS program may be expected to promote prioritization of motor performance at the expense of cognitive performance. For C-Mill therapy, however, it remains an open question which of the tasks will be promoted. On the one hand, one could expect prioritization of cognitive-task performance, considering the nature of the projector-augmented context without physical repercussions of failed adaptive walking. On the other hand, C-Mill therapy takes place on a treadmill with a fixed speed, which is likely to promote prioritization of the motor task over the cognitive task because of time-pressure demands, akin to the posture-first principle observed previously [8, 9]. Regardless of the precise outcomes, the present results clearly underscore the importance of quantifying (changes in) task prioritization for different treatment environments.

In conclusion, by varying the environment we were able to successfully influence the amount of cognitive-motor interference and to induce differences in task prioritization in dual-task walking in people with stroke. Unlike previous studies [8, 9], we did so without constraining walking speed, because we expected and indeed found speed adaptations with dual-tasking and more challenging walking environments, thereby confirming the importance of not constraining walking speed in studies on cognitive-motor interference and task prioritization in people with stroke. Cognitive-motor interference increased for dual-task walking in challenging walking environments. People with stroke seem to differentially prioritize motor and cognitive tasks in the two different challenging walking environments, related to an apparent difference in the consequence of failed adaptive walking as well as differences in the time pressure under which step adjustments are needed to be made and the amount of clutter, placing different demands on attentionally costly walking adaptations and task switching.

## Figures and Tables

**Table 1:** Statistical effects of environment and dual-tasking on walking speed, walking-adaptability performance score, and cognitive-task performance score (mean ± sd). Significant effects are represented in bold and tendencies in bold italic.

	Single-task reference condition	Walking environment			Repeated measures ANOVA		
		Plain	Challenging-physical	Challenging-projected	Environment	Dual-tasking	Environment × dual-tasking
Walking speed (m/s)							
Single		0.90 ± 0.28	0.58 ± 0.20	0.76 ± 0.25	**F**(1.38,40.15) **= 88.90, p** < .01**, partial η**^2^ = 0.75	**F**(1,29) = 34.73, **p** < .01, **partial η**^2^ = 0.55	**F**(2,58) = 5.04, **p** < .05, **partial η**^2^ = 0.15
Dual		0.76 ± 0.24	0.52 ± 0.22	0.67 ± 0.23			
Walking-adaptability performance score (0–10)							
Single			5.38 ± 2.14	6.05 ± 3.45	*F*(1,29) = 0.38, *p* < .55, partial *η*^2^ = 0.01	**F**(1,29) = 11.74, **p** < .01, **partial η**^2^ = 0.91	***F(1, 29) = 3.68, p = .07***, ***partial n***^***2***^***= 0.56***
Dual			4.93 ± 2.02	4.83 ± 3.31			
Cognitive-task performance score (*n*/s)	0.47 ± 0.21	0.48 ± 0.25	0.35 ± 0.21	0.41 ± 0.24	**F**(2,58) = 12.11, **p** < .01, **partial η**^2^ = 0.30		
